# Food and water security issues in Russia II: Water security in general population of Russian Arctic, Siberia and Far East, 2000–2011

**DOI:** 10.3402/ijch.v72i0.22646

**Published:** 2013-12-09

**Authors:** Alexey A. Dudarev, Eugenia V. Dushkina, Yuliya N. Sladkova, Pavel R. Alloyarov, Valery S. Chupakhin, Vitaliy M. Dorofeyev, Tatjana A. Kolesnikova, Kirill B. Fridman, Birgitta Evengard, Lena M. Nilsson

**Affiliations:** 1 Northwest Public Health Research Center, St. Petersburg, Russia; 2 Dubna City Hospital, Moscow Oblast, Russia; 3 Division of Infectious Diseases, Department of Clinical Microbiology, Umeå University, Umeå, Sweden; 4 Arcum, Arctic Research Centre at Umeå University, Umeå, Sweden; 5 Department of Public Health and Clinical Medicine, Nutritional Research, Umeå University, Umeå, Sweden

**Keywords:** water security, drinking water, centralized, non-centralized water sources, chemical, biological contamination, pollutants, bacteria, spores, cysts, virus, pesticides, metals, Russian Arctic

## Abstract

**Background:**

Poor state of water supply systems, shortage of water purification facilities and disinfection systems, low quality of drinking water generally in Russia and particularly in the regions of the Russian Arctic, Siberia and Far East have been defined in the literature. However, no standard protocol of water security assessment has been used in the majority of studies.

**Study design and methods:**

Uniform water security indicators collected from Russian official statistical sources for the period 2000–2011 were used for comparison for 18 selected regions in the Russian Arctic, Siberia and Far East. The following indicators of water security were analyzed: water consumption, chemical and biological contamination of water reservoirs of Categories I and II of water sources (centralized – underground and surface, and non-centralized) and of drinking water.

**Results:**

Water consumption in selected regions fluctuated from 125 to 340 L/person/day. Centralized water sources (both underground and surface sources) are highly contaminated by chemicals (up to 40–80%) and biological agents (up to 55% in some regions), mainly due to surface water sources. Underground water sources show relatively low levels of biological contamination, while chemical contamination is high due to additional water contamination during water treatment and transportation in pipelines. Non-centralized water sources are highly contaminated (both chemically and biologically) in 32–90% of samples analyzed. Very high levels of chemical contamination of drinking water (up to 51%) were detected in many regions, mainly in the north-western part of the Russian Arctic. Biological contamination of drinking water was generally much lower (2.5–12%) everywhere except Evenki AO (27%), and general and thermotolerant coliform bacteria predominated in drinking water samples from all regions (up to 17.5 and 12.5%, correspondingly). The presence of other agents was much lower: Coliphages – 0.2–2.7%, Clostridia spores, Giardia cysts, pathogenic bacteria, Rotavirus – up to 0.8%. Of a total of 56 chemical pollutants analyzed in water samples from centralized water supply systems, 32 pollutants were found to be in excess of hygienic limits, with the predominant pollutants being Fe (up to 55%), Cl (up to 57%), Al (up to 43%) and Mn (up to 45%).

**Conclusion:**

In 18 selected regions of the Russian Arctic, Siberia and Far East Category I and II water reservoirs, water sources (centralized – underground, surface; non-centralized) and drinking water are highly contaminated by chemical and biological agents. Full-scale reform of the Russian water industry and water security system is urgently needed, especially in selected regions.

Although available freshwater resources in the Arctic rank among the largest in the world (1), quality and quantity of household water may differ substantially among countries and regions. Thus, water security has been highlighted as a prioritized issue in the Arctic, and 6 indicators of water security (including quality aspects) have recently been promoted for international comparisons. These are per capita renewable water, accessibility of running water, waterborne diseases and contaminants in drinking water, authorized water quality assurance and the existence of water safety plans (2).

From a Russian perspective, the issue of drinking water-related contaminants is an urgent matter. About 70% of the population of the Russian Federation obtains drinking water from surface water sources, 40% of which do not comply with hygienic norms, which includes aspects that may be categorized as either sanitary or aesthetic norms from a western perspective. More than 27% of water pipelines from surface reservoirs are not equipped with water purification facilities and 16% lack disinfection systems (3).

Permafrost which occupies about 65% of Russian territory (including the whole Arctic, and the bulk of Siberia and Far East) is the main cause of infrequent use of underground water sources in the northern territories of Russia. In small settlements, as a rule, water pipes supply untreated and non-disinfected drinking water directly from surface water sources. A majority of these water supply systems in rural areas are used only in summer. In winter, water for household needs and drinking is mostly delivered from surrounding, often unexplored, reservoirs due to the insufficient flow rates of open water sources. Some communities have “technical” pipeline water-delivery systems from the nearest lake or river, and use constant water preheating during cold seasons, which serves as a centralized combination of house heating and hot-water supply, to avoid water freezing. In this situation, pure cold drinking water is not provided. In severe cold climate zones where wells are unavailable or impossible to construct, water will typically be delivered by trucks carrying water tanks in summer and sawn ice blocks in winter.

In rural areas, more than one-third of the population uses drinking water from non-centralized sources. The quality of this water is low due to weak protection of aquifers from pollution from surface areas, the lack of sanitary protection zones, and the delayed repair, cleaning and disinfecting of wells and interception ditches. Almost everywhere, municipal financing for these purposes is simply not appropriated (3, 4).

Of particular concern from a water contaminant perspective is the serious deterioration of water distribution and sewerage networks, and the numerous accidents on these networks that leads to secondary pollution of drinking water. In recent years, the systematic preventive maintenance and repair of water supply facilities and networks have been almost completely replaced by recovery efforts after accidents. Currently in Russia, about one-third of the water-supply and sewerage networks have deterioration levels of more than 60%. Restoration of these systems (bringing them to the proper sanitary condition) will take more than 50 years, based on the current rate of repair (3).

In the Russian Federation, drinking water from centralized water supplies that do not meet hygienic standards for chemical substances is consumed by more than 10 million people, and it supplies that do not meet the standard for indicator bacteria, by more than 14 million people annually. In 2006–2007, conditionally pathogenic and pathogenic microorganisms in drinking water have been recorded in 56 administrative Russian territories. Outbreaks of gastroenteric infectious diseases, including hepatitis, are often caused by microbial contamination of drinking water (5).

About 28% of the Russian population consume highly mineralized drinking water (1.6–10 g/L), which promotes the risk of cardiovascular diseases, urolithiasis, and so on. About 85 million people consume water with low fluorine content (2–5 times lower than recommended), which leads to a 90–100% prevalence of caries among children in some regions. About 50 million people in the country (one-third of the population) consume drinking water with enhanced iron content (5, 6).

The poor state of water supply systems owned by the Russian state, and the poor quality of drinking water is publicly admitted, particularly regarding the regions of the Russian Arctic, Siberia and Far East (3–6). Despite this, the Russian Federation still has no federal law on drinking water and drinking water supply. Such a law was elaborated and submitted for consideration 14 years ago and adopted by the State of Duma in December 1999, but after that, it was immediately rejected by the upper chamber of Russian Parliament mainly due to disagreements with regard to the regulations governing the mechanism for the privatization of drinking water supply systems (Information on Causes of rejection of Federal law on “Drinking water and drinking water supply” of the Federation Council of the Russian Federation, http://base.consultant.ru).

In 2006, the governing party “United Russia” initiated an all-Russia large-scale clean water project that includes investments in a unique universal “Golden Formula” nanotech water filter, known as Petrick-Gryzlov filters; these “filters” were said to be able to block any pollutants in drinking water, including radioactivity (7). When this claim was proven to be false (7, 8), the project was reviewed, and in December 2010 the Federal Target Program “Clean water” for the period 2011–2017 was adopted by the Government (9). The total budget of the Program for 7 years was set at 331.8 billion roubles (about 10 billion USD).

On 1 January 2013, the Federal Law “On water supply and water outlet” was entered into force. This law regulates only economic and financial issues. Regional target programmes aimed at providing high-quality drinking water are operating in 33 Russian regions (among them Murmansk Oblast, Karelia, Khanty-Mansy AO, Krasnojarsk kraj, Magadan Oblast, Primorsky kraj, Chukotka); but financing of some of these programmes has been “insufficient” or “not approved” (5).

Regions of the Russian Arctic, Siberia and the Far East where the sanitary–chemical indicators of drinking water quality did not meet hygienic requirements (more than 1.5 times the limit) include Kareliya Republic, Arkhangelsk Oblast, Nenets AO, Yamalo-Nenets AO, Khanty-Mansi AO, Yakutia Republic, Chukotka AO, Sakhalin oblast, and regions where the microbiological indicators of drinking water quality did not meet hygienic requirements (more than 1.5 times the limit) include Kareliya Republic, Arkhangelsk oblast, Yakutia Republic, Sakhalin oblast, Khabarovsk kraj, Primorsky kraj and Amur Oblast (5).

The most comprehensive assessment of all aspects of water supply and water quality (including chemical and biological contamination issues) in the Russian northern regions has been carried out in several studies in Arkhangelsk city and Arkhangelsk oblast (10–16). Several studies carried out on water security have reported a great many problems in other regions: Khanty-Mancy AO (17, 18), Krasnojarsk kraj (19), Yakutia (20, 21) and Primorsky kraj (22). Bacterial and viral contamination of waters in the Eastern Siberian region (Krasnojarsk kraj, Yakutia, etc.) have been investigated by the Irkutsk Research Institute of Epidemiology and Microbiology (23–25) where results on Hepatitis A, Cytomegalovirus and Rotavirus in drinking water are of particular interest. The presence of the parasitic protozoan, *Giardia lamblia*, in drinking water and even in bottled water has been reported in several cities and settlements of Yakutia (20).

This study is the first complex comparative assessment of water quality (including drinking water-related chemical and biological contaminants) in the regions of the Russian Arctic, Siberia and Far East, which uses unified water security indicators collected from statistical sources.

## Objectives

Our general aim was to compare water security indicators (including chemical and biological contaminants in drinking water) in 18 regions of the Russian Arctic, Siberia and Far East (for the period 2000–2011) and to assess water safety in these territories.

## Study design and methods

Eighteen regions of the Russian north, Siberia and Far East (see “Food and water security issues in Russia I: food security …” in the current volume of IJCH) have been included in the study, and the following official statistical data sources were used (for the period 2000–2011):

- Regional Statistical Yearbooks (trade statistics) – all regions except Khanty-Mansi AO, Taymyr AO, Evenki AO, Koryak AO and Sakhalin Oblast.
- Regional State Reports on “Sanitary–epidemiological situation” (excesses in percentages above national hygienic limits of chemical and biological water contamination) – all regions except Taymyr AO, Evenki AO, Koryak AO and Primorsky kraj.
- Federal Automatic system “Social–Hygienic Monitoring” (data on specific biological and chemical contaminants in different water sources and in drinking water) – all regions except Koryak AO.

The following water safety data have been analyzed in selected regions:

- Water consumption (L/person/day)
- Chemical and biological contamination of water reservoirs of Categories I and II
- Chemical and biological contamination of water sources (centralized – underground and surface, as well as non-centralized) and drinking water
- Specific chemical and biological contaminants in drinking water

### Specification of Russian hygienic regulations of water contamination

All water reservoirs in Russia are divided into 2 categories. The first category includes bodies of water used for drinking and household water use, as well as for water supply used for the food industry. The second category includes bodies of water for recreational use. Water quality requirements set for the second category of water use also apply to all areas of bodies of water that are within the boundaries of built-up areas.

A set of sanitary rules and norms (Hygienic requirements for surface water) (26) regulates water quality of bodies of water used for drinking, household and recreational uses, conditions of wastewater discharge into water bodies, requirements relating to placement, design, construction, renovation and exploitation of industrial and other objects that may have an impact on surface water, as well as requirements for the organization of the monitoring of water quality of water bodies.

Another set of sanitary rules and norms (Drinking water: Hygiene requirements regarding the quality of centralized water supply systems – Quality Control) (27)is the main document in Russia that regulates the safety of drinking water in terms of chemical, biological and radioactive contamination with addenda on requirements to materials, reagents, equipment used for water purification and treatment (28). Important additional documents include “Maximum permissible concentrations (MPC) of chemical substances in bodies of water used for drinking, household, and cultural and community water uses” (29) and “Approximate permissible concentrations (APC) of chemicals in bodies of water used for drinking, household, cultural and community water uses” (30).

In accordance with the hygienic rules, the sanitary–chemical quality of water is assessed according to several criteria, including organoleptic properties (colour, smell, taste and suspended matter), pH (mineralization, hardness and oxidability), oil and oil products, surfactant species, phenol index, and non-organic and organic chemical substances.

According to the “Social–hygienic monitoring” Federal Information system for 2003–2007, the prioritized pollutants in drinking water for centralized water pipelines are due to water source contamination, water contamination during water treatment and water contamination during water transport in pipelines (5).

Examples of Russian national threshold levels for some pollutants in drinking water (mg/L) are: Hg – 0.0005; Pb – 0.03; Cd – 0.001; HCH – 0.002; DDT – 0.002.

When we evaluate data on chemical contamination of water, we must bear in mind that values such as “excess percentage over hygienic threshold” are actually combinatory hygienic appraisals that can be attributed not only to chemical substances but also to organoleptic properties, such as colour, pH, mineralization and hardness.

Microbiological quality of water is assessed according to several criteria (27): total bacterial count (heterotrophic plate count) should not be more than 50 CFU/ml of water; general and thermotolerant Coliform bacteria, Coliphages, sulphite-reducing Clostridia spores and/or Giardia cysts are not permitted.

Isolation and identification of specific pathogenic microorganisms in water is a complicated and expensive task. As searches for pathogens in water are usually substituted by the assessment of some indicator microorganisms, so the monitoring and control of microbiological water contamination is an indirect process. As it is generally considered that microbiological contamination occurs mostly by faecal waste water, the small group of non-pathogenic organisms as indicators of faecal excretion of humans and animals has been selected. These microorganisms could be isolated and identified with relative ease. They have similar (to pathogens) origin and viability, presented in water in much higher (than pathogens) quantities, and they can serve as a sufficiently reliable indicator of faecal water contamination. Despite certain shortcomings of the indirect method of assessment of contamination of water (31), this approach is applied everywhere in Russia.

Coli bacteria are able to survive in water for several weeks and are easily identified – in Russia they are the main indicators. Sulphite-reducing Clostridia (and particularly their spores) in water can exist infinitely—it is very tolerable to environmental factors. The presence of clostridia spores in water indicates long-standing faecal pollution; this agent is particularly useful when testing open water, as it gives an indication of the presence of microorganisms resistant to disinfectants (32). Giardia cysts are indicators of protozoa organisms in water, Coliphages – of enteroviruses (human enteric viruses). Depending on the time of travel between source and recipient, the relative number of *Cryptosporidium hominis* oocysts rapidly increases, particularly in warmer (summer-time) waters. Hence, *Giardia lamblia* may not be the best index for the presence of other parasitic protozoa, but certainly worthwhile measuring, and as both *Giardia* and *Cryptosporidium* spp. are generally assayed together (33). Thus, if the presence of indicator organisms in water has been revealed, it is necessary to assume the presence of pathogenic agents also.

### Measurement of biological and chemical contaminants in drinking water

#### Biological contaminants

The collection of data from “Social–Hygienic Monitoring” Federal Automatic system is estimated at about 378,000 analyzed water samples for biological contaminants from all selected regions during 2007–2011. This database has enabled us to evaluate selected biological contaminants which are monitored in the regions. Total numbers of water samples analyzed in selected regions were very different and varied from 360 to 10,000 analyses averaged per year; being adjusted to the population number of each region (per 10,000 population) to make the results more comparable – from 30 to 122 per 10,000/year with the exception of Chukotka (294 per 10,000/year), which shows the highest sampling frequency, and Khabarovsk kraj (6.7 per 10,000/year), which has the most poorly performing food contaminants laboratory monitoring of all the regions.

#### Chemical pollutants

In contrast to the biological water contaminants data array, the “Social–Hygienic Monitoring”, the Federal Automatic system does not possess data on the number of samples analyzed for chemical pollutants (totally or with regard to specific pollutants) in the regions. However, information on concentrations of selected water pollutants that do not comply with hygienic norms is available. This database has enabled us to evaluate 2006–2011 selected chemical pollutants that are monitored by the regions.

A total of 56 chemical pollutants were analyzed in water samples from centralized water supply systems in all selected regions during the period specified, namely mercury, lead, selenium, ammonia and ammonium ion, strontium, sulphates, sulphides and hydrogen sulphide, carbon tetrachloride, trichloromethane, barium, formaldehyde, fluorine 1–2, fluorine 3, chlorine, chlorides, chromium (+3), chromium (+6), cyanide, zinc, ethylbenzole, benzol, beryllium, boron, 2,4-D, HCH, HCB, phenol, aluminium, aluminium chloride hydroxide, iron, ferric chloride, iodine, cadmium, potassium silicate, calcium phosphate, cobalt, silicon, lithium, magnesium, manganese, copper, methane acid, methanol, methylbenzene, molybdenum, arsenic, sodium, oil, sulphur oil, nickel, nitrates, nitrites, nitrobenzene, polyacrylamide, polyphosphates, tetrachlorethylene and trichloroethylene.

## Results

Data on annual per capita water consumption from centralized water sources are available for a majority of the regions, see Table I (data collected from (34, 35). In the selected regions, water consumption fluctuates from 125 to 340 L/person/day, which can be compared with the Russian average consumption of 237 L/person/day. The highest values were reported from Magadan Oblast, Kamchatka, Khabarovsk kraj and Yamalo-Nenets AO, and the lowest from Nenents AO, and predominantly (less than 125 L) from Chukotka and Yakutia.

**Table I:** Water consumption in selected regions (centralized water sources) in L/person/day

	Years	L/person/day
Russian Federation	2000–09	237.3
Murmansk Oblast	nd	nd
Karelia Republic	nd	nd
Arkhangelsk Oblast	2004–08	239.7
Nenets AO	2006–10	167.9
Komi Republic	2000–09	237.5
Yamalo-Nenets AO	2000–09	276.7
Khanty-Mansi AO	2008–09	260.3
Taymyr AO	nd	nd
Evenki AO	nd	nd
Yakutia Republic	2003–10	124.7
Magadan Oblast	2000–08	340
Koryak AO	nd	nd
Chukotka AO	2011	125.0*
Kamchatka kraj	2000–08	303.6
Sakhalin Oblast	nd	nd
Khabarovsk kraj	2006–10	287.7
Primorsky kraj	2006–10	224.1
Amur Oblast	2002–10	177

Sources: Regional Statistical Yearbooks (34, 35).

* From the State report “On sanitary-epidemiological situation in Chukotka AO, 2011” (36). nd, no data.

Chemical and biological contamination of water reservoirs of Categories I and II are presented in Table II (data collected from 36, 37). In general, both categories of water reservoirs are contaminated to a similar extent with regard to all types of pollutants. In some regions, the extent of contamination of drinking water sources (Category I) could be higher than that of recreational waters (Category II). This is also true for Russia, as a whole, where 22–27% of samples from all water objects are contaminated.

**Table II:** Chemical and biological contamination of water reservoirs of Categories I and II, percentage of water samples that do not comply with hygienic norms

		Category I		Category II	
			
	Years	Chemical	Biological	Chemical	Biological
Russian Federation	2002–10	27.5	21.9	26.4	23.4
Murmansk Oblast	2007–09	32.5	2	37.3	8.5
Karelia Republic	2003–11	26.8	13.5	38.2	26.7
Arkhangelsk Oblast	2007–11	59.3	31.5	39.7	51.3
Nenets AO	2009–11	59.2	30	48.6	23.7
Komi Republic	2002–11	40	8.3	16	21.6
Yamalo-Nenets AO	2007–11	54.9	22.2	46.3	12.4
Khanty-Mansi AO	2006–11	80.2	21.4	nd	25.9
Taymyr AO	2006–08	3.8	2.0	nd	nd
Evenki AO	2006–08	13.9	38.1	nd	nd
Yakutia Republic	2002–11	39.9	26.4	33.5	34.6
Magadan Oblast	2006–10	37.7	9.3	nd	nd
Koryak AO	nd	nd	nd	nd	nd
Chukotka AO	nd	nd	nd	nd	nd
Kamchatka kraj	2007–11	10.2	8.2	6.5	28.9
Sakhalin Oblast	2001–11	15.3	9.9	21.1	23.9
Khabarovsk kraj	2005–11	15.2	43.1	9.5	60.9
Primorsky kraj	nd	nd	nd	nd	nd
Amur Oblast	2003–11	21.3	26.6	32.6	37.6

Sources: Regional State reports “On sanitary-epidemiological situation” (36, 37) and (5). nd, no data.

The worst water quality regarding both sanitary–chemical and biological contamination concerning both categories of water objects was reported from Arkhangelsk Oblast, Nenets AO. These indices are about twice as high as the Russian average. In addition, high levels of chemical contamination of Category 1 water reservoirs could be seen in Khanty-Mancy AO (80%), Yamalo-Nenets AO (54.9%), Komi Republic (40.0%), Yakitia Republic (39.9%) and Magadan Oblast (37.7%); high levels of chemical contamination of Category II water reservoirs are found in Murmansk Oblast (37%), Karelia (38%) and Yamalo-Nenets AO (46%). Biological contamination of both categories of water objects is generally substantially lower than chemical contamination, and especially lower compared to average Russian levels. Here, the “leaders” of Category 1 reservoirs are Khabarovsk kraj (43%), Evenki AO (38.1%) and the “worst pair,” Arkhangelsk Oblast (31.5%) and Nenets AO (30.0%), and in the case of Category 1I reservoirs, Khabarovsk kraj (61%), Arkhangelsk Oblast (51%), Yakutia (35%) and Amur Oblast (38%).

Chemical and biological contamination of water sources (centralized, divided into underground and surface, and non-centralized) is presented in Table III (data collected from 36, 37).

**Table III:** Chemical and biological contamination of water sources (centralized – underground, surface, and non-centralized), percentage of water samples that do not comply with hygienic norms

		Water sources							
		
		Centralized		Underground		Surface		Non-centralized	
					
	Years	Chemical	Biological	Chemical	Biological	Chemical	Biological	Chemical	Biological
Russian Federation	2005–10	28	6.5	28.4	4.9	25.4	18.3	27.4	23.9
Murmansk Oblast	2002–11	27	1.6	35.8	0.5	28.9	1.8	20	7.4
Karelia Republic	2005–11	25.2	8.8	34.9	5.6	15.9	5	26.7	34
Arkhangelsk Oblast	2002–11	51	19	nd	nd	73.5	35.4	43.5	48.1
Nenets AO	2005–11	24.7	6.2	nd	nd	nd	nd	37	14.7
Komi Republic	2002–11	42.5	2.9	54.4	1.9	48.2	6.8	45.1	34.4
Yamalo-Nenets AO	2005–11	57.2	nd	64.3	1.9	61.9	27.5	nd	nd
Khanty-Mansi AO	2005–11	79.1	nd	76.2	1.2	79.5	4.8	nd	nd
Taymyr AO	2006–11	25.9	5.1	nd	nd	nd	nd	54.2	4.6
Evenki AO	2007–11	40.7	54.5	nd	nd	nd	nd	25.8	31.9
Yakutia Republic	2002–11	17.6	11.9	15.4	17.8	25.5	19.7	37.3	27.8
Magadan Oblast	2002–11	24.1	5.1	19	2.6	nd	nd	11.1	13.6
Koryak AO	nd	nd	nd	nd	nd	nd	nd	nd	nd
Chukotka AO	2005–11	33	5.5	13.4	1.8	48.8	11.9	90*	10.3
Kamchatka kraj	2008–11	nd	nd	3.9	2.6	8.4	6.5	8.8	8.6
Sakhalin Oblast	2001–11	24.6	6.1	nd	nd	19.2	7.1	14.5	23.6
Khabarovsk kraj	2007–11	nd	12.6	25.7	7	15.2	43	28.1	24.3
Primorsky kraj	nd	nd	10.7	nd	nd	nd	nd	nd	nd
Amur Oblast	2004–11	24.1	7.1	19.9	3.2	11.1	0.2	26.9	25.4

Source: Regional State reports “On sanitary-epidemiological situation” (36, 37). nd, no data.

* Data available for 2011 only.

Though centralized water sources (both underground and surface sources) are highly contaminated by chemicals throughout Russia (25–28%), contamination is even higher (40–80%) in Arkhangelsk Oblast, Komi Republic, Yamalo-Nenets AO, Khanty-Mancy AO and Evenki AO. Similarly, biological contamination is much higher than the national average (up to 55%, as compared to 5–18%) in Arkhangelsk Oblast, Yamalo-Nenets AO, Khanty-Mancy AO, Evenki AO, Yakutia and Khabarovsk kraj. Biological contamination of centralized water sources is mostly represented by surface waters. Underground water sources show relatively low levels of biological contamination, while chemical contamination of both sources is relatively similar, a situation that could be caused by additional water contamination during water treatment and transport through pipelines. Non-centralized water sources are highly polluted in Arkhangelsk Oblast and Komi republic (35–48% – both chemical and biological), Nenets AO, Taymir AO, Yakutia and Chukotka (37–90% – chemical), and Evenki AO (biological – 32%).

**Fig. 1:**
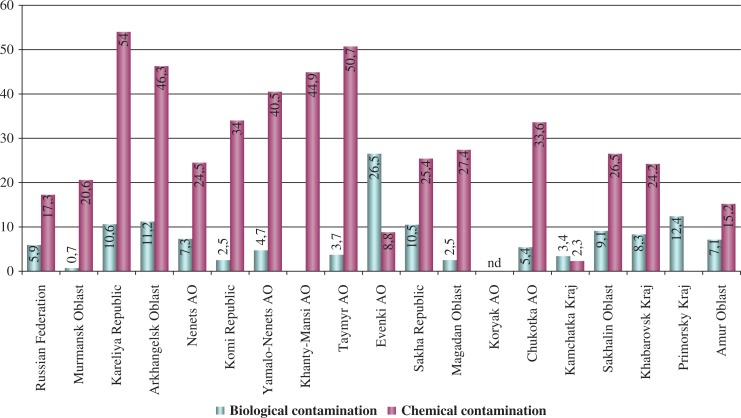
Chemical and biological contamination of drinking water (running water), percentage of water samples the do not comply with hygienic norms. Data from (36, 37).

Chemical and biological contamination of drinking water (tap water) is presented in Fig. 1. Very high levels of chemical contamination of drinking water (up to 51%) are obvious in many regions, mainly in the north-western part of the Russian Arctic (from Karelia to Taymir). Biological contamination is much lower (2.5–12%) everywhere else, with the exception of Evenki AO (27%).

### Biological contaminants in water

**Fig. 2:**
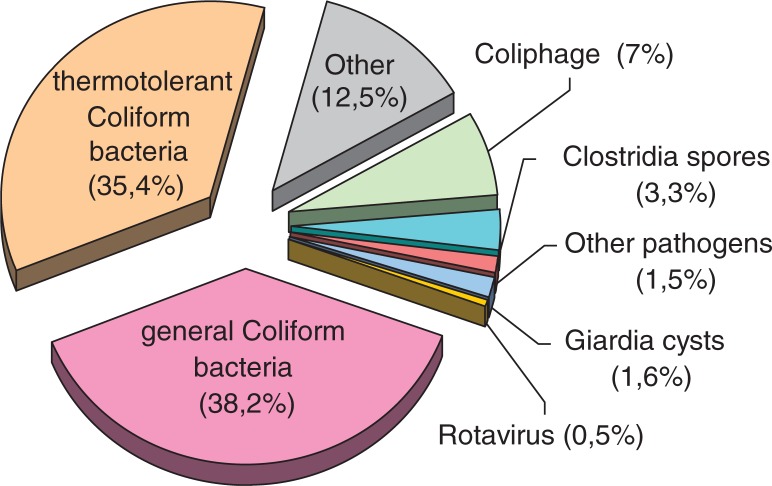
Distribution of biological contaminants in all water samples from all selected regions (2007–11), percentage of total number of samples analyzed.

The total distribution of these biological contaminants is presented in Fig. 2. A total of 87.5% of water samples (Fig. 2) have been analyzed for 7 contaminants – general and thermotolerant Coliform bacteria (74%), Coliphages (7%), sulphite-reducing Clostridia spores (3.3%), Giardia cysts (1.6%), Rotavirus (0.5%) and other pathogens (1.5%).

Table IV presents the number of water samples analyzed for all biological contaminants and separately, for 7 main contaminants in each selected region, on average during the specified period, and percentage of samples that exceeded Russian hygienic thresholds.

**Table IV:** Number of drinking water samples analyzed for all biological agents and specific contaminants in selected regions, on average, during specified periods, number of samples and percentage of samples where biological agents have been detected

		All biological contaminants		Total Coliform bacteria		Thermo tolerant Coliform		Coliphage		Clostridia spores	
							
	Years	*n* per 10,000 population	*n*	*n*	%	*n*	%	*n*	%	*n*	%
Murmansk Oblast	2007–11	104.6	8,676	3,343	1.3	3,328	1.2	749	2.3	84	0
Karelia Republic	2007–11	55.5	3,740	1,468	8	1,425	4.2	285	2.4	88	0
Arkhangelsk Oblast	2007–11	54	6,758	1,984	7.8	1,952	5.1	863	2.7	404	0.1
Nenets AO	2010–11	86.5	364	75	2.3	75	1	109	0	105	0
Komi Republic	2007–11	107.1	10,085	3,266	1.5	2,978	1	687	0.03	352	0.2
Yamalo-Nenets AO	2007–11	122.3	6,594	2,646	4.9	2,653	3.2	95	0.3	412	0
Khanty-Mansi AO	2007–11	75.1	11,458	4,745	2	4,944	1.1	539	0.6	246	0.1
Taymyr AO	nd	nd	nd	nd	nd	nd	nd	nd	nd	nd	nd
Evenki AO	nd	nd	nd	nd	nd	nd	nd	nd	nd	nd	nd
Yakutia Republic	2007–11	92.8	8,838	2,861	8.2	2,238	5.5	463	0.2	471	0.04
Magadan Oblast	2007–11	59.4	960	417	1.3	344	0.9	115	0.4	ns	ns
Koryak AO	nd	nd	nd	nd	nd	nd	nd	nd	nd	nd	nd
Chukotka AO	2008–11	293.9	1,467	620	3.7	596	2.2	61	1.2	31	0.8
Kamchatka kraj	2007–11	114.9	3,899	1,890	3.4	1,772	2.3	100	0.4	14	0
Sakhalin Oblast	2007–11	108.2	5,515	2,221	9	1,751	6.4	471	0.8	366	0
Khabarovsk kraj	2007–11	6.7	928	317	3.2	260	1.5	203	0.2	ns	ns
Primorsky kraj	2007–11	29.7	5,883	2,709	17.5	2,356	12.1	685	2.7	1	0
Amur Oblast	2007–11	29.9	2,556	1,086	3.7	869	3.7	36	0	ns	ns
				Giardia cysts		Pathogenic bacteria		Rotavirus		Other biological	
							
				*n*	%	*n*	%	*n*	%	*n*	%

Murmansk Oblast	2007–11			316	0	12	0	ns	ns	844	0
Kareliya Republic	2007–11			48	0.4	45	0	128	1.6	253	0.9
Arkhangelsk Oblast	2007–11			295	0.1	163	0	2	0	1,095	0.7
Nenets AO	2010–11			ns	ns	ns	ns	ns	ns	ns	ns
Komi Republic	2007–11			65	0	ns	ns	ns	ns	2,736	0.1
Yamalo-Nenets AO	2007–11			56	0	148	0	101	31.2	482	3.1
Khanty-Mansi AO	2007–11			26	0	24	0	26	0	908	0.9
Taymyr AO	nd			nd	nd	nd	nd	nd	nd	nd	nd
Evenki AO	nd			nd	nd	nd	nd	nd	nd	nd	nd
Yakutia Republic	2007–11			192	0	388	0.1	ns	ns	2,224	2
Magadan Oblast	2007–11			ns	ns	ns	ns	67	0	17	0
Koryak AO	nd			nd	nd	nd	nd	nd	nd	nd	nd
Chukotka AO	2008–11			10	0	10	0	ns	ns	139	0.9
Kamchatka kraj	2007–11			22	0	18	0	ns	ns	83	0
Sakhalin Oblast	2007–11			60	0	69	0	2	0	575	0.8
Khabarovsk kraj	2007–11			71	0	14	0	ns	ns	65	0
Primorsky kraj	2007–11			89	0	15	0	27	2.8	ns	ns
Amur Oblast	2007–11			12	0	240	0	12	8.3	301	3.9

Source: “Social-hygienic monitoring” system.

n, average number of samples per year; nd, no data; ns, no samples analyzed; %, of samples where biological agents have been detected.

With regard to the 7 main contaminants in water samples (Table IV), general and thermotolerant Coliform bacteria and Coliphages were assessed for all selected regions. Clostridia spores have been analyzed in all regions except Magadan Oblast, Khabarovsk kraj and Amur Oblast. Giardia cysts and pathogenic bacteria are not monitored in Nenets AO and Magadan Oblast. In Komi republic, pathogenic bacteria and Rotavirus have not been analyzed. It is important to emphasize that Rotavirus is being monitored in only half of the regions studied.

As for the percentage of samples that do not comply with hygienic norms (Table IV), general and thermotolerant Coliform bacteria predominate in all regions (from 1 to 17.5%, with Primorsky kraj showing the highest values). The presence of Coliphages was not high in any region (0.2–2.7%). The presence of Clostridia spores, Giardia cysts and pathogenic bacteria did not exceed 0.8% (Clostridia in Chukotka) and was almost near zero. Rotavirus has been detected in water samples in 4 regions with the highest value of 31% in Yamalo-Nenets AO. We must remember that according to hygienic norms, the presence in drinking water of any of the biological agents listed above is not permitted. According to sanitary rules and norms (22), the “necessity of water analysis for pathogenic enteric bacteria or enteroviruses is determined by epidemiologic indications, and should be prescribed by a Sanitary Inspection Center.”

### Chemical contaminants in water

A total of 56 chemical pollutants (during specified period) have been analyzed in water samples from centralized water supply systems (from all selected regions). Among them the excess of hygienic limits were set on 32 chemical pollutants; 9 of them (main pollutants which concentrations over hygienic limits in water samples were recorded most frequently) are presented in Table V.

**Table V:** Main chemical water pollutants in samples from centralized water supply systems in selected regions, percentage of samples that do not comply with hygienic norms (averaged for specified periods)

	Years	Fe	Mn	Al	Cl	Chlorides	Sulphates	Nitrates	Nitrites	Ammonia
Murmansk Oblast	2006–11	24.2	4.6	16.2	5.2	3.6	ns	2.2	2.1	0.2
Karelia Republic	2006–11	30.4	8.8	1.3	2.1	0	0	0	0	1
Arkhangelsk Oblast	2006–11	28.9	4	43.2	57.1	0	0.9	0.5	0	0.9
Nenets AO	2006–11	3.3	ns	ns	ns	0	0	0	0	0
Komi Republic	2006–11	36.9	19.4	18.4	2.6	0.5	0.9	0.5	0.6	6.2
Yamalo-Nenets AO	2006–11	36.9	20.8	0	ns	5.9	0	0	0	1.1
Khanty-Mansi AO	2006–11	54.8	45.1	0.1	0.1	6.5	5.9	5.6	4.8	24.1
Taymyr AO	2006–11	0	ns	ns	ns	0	0	0	0	0
Evenki AO	nd	nd	nd	nd	nd	nd	nd	nd	nd	nd
Yakutia Republic	2006–11	12.6	0.3	0	22.2	4.6	1.2	0	6.7	8.2
Magadan Oblast	2006–11	2.7	9.7	nd	0	0	0	0	0	3.7
Koryak AO	nd	nd	nd	nd	nd	nd	nd	nd	nd	nd
Chukotka AO	2006–11	27.7	10.4	0	0	0.7	11.6	14.8	18.8	18.8
Kamchatka kraj	2006–11	1.7	0.5	0	0	6.4	2.7	1	0	0
Sakhalin Oblast	2006–11	13.4	9.7	0	7.2	0	0	0	0	1.2
Khabarovsk kraj	2006–11	26.6	8.9	0	nd	0	0.7	1.6	0	0.1
Primorsky kraj	2006–11	18.3	4.5	0.4	ns	0	0	0	0	0.4
Amur Oblast	2006–11	13.1	10.3	9.1	0	0	0	6	6.4	6.4

Source: “Social-hygienic monitoring” system.

nd, no data; ns, no samples analyzed.

All 9 pollutants were assessed in all selected regions (Table V) with the exception of manganese, aluminium and chlorine in Nenets AO and Taymir AO. The results for these 2 regions look very strange as no excess of hygienic limits of the pollutants in water samples has been detected except for a slight rise of iron in Nenets AO. In other words, it would seem that data presented here are untrustworthy. Additionally, chlorine has not been analyzed in Yamalo-Nenets AO and Primorsky kraj, and sulphates in Murmansk Oblast.

As for the percentage of samples that exceeded the Russian hygienic threshold (Table V), iron dominated in all regions (from 2–3% in Kamchatka and Magadan Oblast to 55% in Khanty-Mancy AO). Excesses of manganese significantly fluctuate (from 0.3–0.5% in Kamchatka and Yakutia to 45% in Khanty-Mancy AO). Aluminum is high (16–43%) in Murmansk Oblast, Arkhangelsk Oblast and Komi Republic, and chlorine – in Yakutia and Arkhangelsk Oblast (22 and 57%, respectively). Excesses of other pollutants in different regions are episodic, and are generally not high, with the exception of Khanty-Mancy AO and Chukotka, 2 regions that break the records among the selected regions with respect to 4 pollutants: sulphates (6 and 12%, respectively), nitrates (6 and 15%), nitrites (5 and 19%), ammonia (24 and 19%). Obviously, local inspection of water chemical pollutants in these 2 regions function really efficiently, and local hygienic statistics work properly.

Other pollutants (among the 32 registered with excesses) have been analyzed in few regions by sporadic sampling (number of samples unknown). The list below shows examples of the highest percentages of samples that do not comply with hygienic thresholds:

- Mercury in Chukotka in 2010 (38%)
- Cadmium in Khanty-Mansi AO in 2007 (96%)
- Strontium in Arkhangelsk Oblast in 2006 (57%)
- Fluorine in Yamalo-Nenets AO in 2007 (46%)
- Boron in Khanty-Mansi AO in 2007 (100%)
- Nickel in Murmansk Oblast in 2007 (21%),
- Calcium phosphate in Chukotka in 2006 (38%)
- Silicon in Yamalo-Nenets AO in 2007–2008 (97 and 78%), in Khanty-Mansi AO in 2007 (100%), Yakutia Republic in 2008 (100%)
- Magnesium in Yakutia Republic in 2007–2010 (34, 15, 47 and 34%, respectively)
- Copper in Yamalo-Nenets AO in 2011 (23%), in Chukotka in 2006 (14%)
- Tetrachloromethane and trichloromethane in Kareliya Republic in 2009 (39%), in Komi Republic in 2008 (25%)
- Sodium in Yakutia Republic in 2008 (100%)

Extremely high percentages of water samples analyzed for some toxic pollutants (which exceeded hygienic thresholds) have been observed in several regions.

In addition, it is important to emphasize that 3 pesticides (DDT, HCH and 2,4-D) must be regularly monitored in drinking water according to sanitary rules and norms (22). However, no analysis regarding DDT was carried out in the selected regions during the whole period of observation, while analyses results were below threshold limits on 2,4-D in 4 Far Eastern regions (Primorsky kraj, Amur Oblast, Sakhalin and Khabarovsky kraj), on HCH – in the latter regions and additionally, in Arkhangelsk Oblast, Komi Republic, Khanty-Mancy AO and Yakutia.

It is also important to note that in this article, we do not assess waterborne diseases which have surface contact origin such as showering, using humidifiers or toilet flushing (e.g. *Legionella pneumophila* and nontuberculous mycobacteria) (38). These environmental pathogens in Russia are not current in the assessment because no cases of *Legionella pneumophila* or nontuberculous mycobacteria have ever been detected in the studied Russian regions.

## Conclusions

This study, which is based on official statistical data, confirms the poor state of water quality in 18 selected regions of the Russian Arctic, Siberia and Far East for the period 2000–2011. Chemical and biological contamination of water reservoirs of Categories I and II, of water sources (centralized – underground and surface, and non-centralized) and of drinking water in all selected regions is high, and in the majority of regions, very high. In some regions, the extent of contamination of drinking water sources could even be higher than levels found in recreational waters.

In conclusion, our data serve as a good illustration of the alarming water security situation in Russia, where the federal law on “Drinking water and drinking water supply” has still not been approved and a fortiori enforced. Even though the federal Target Program “Clean water” for the period 2011–2017 has been adopted by the Government, regional target programmes aimed at providing high-quality drinking water are “insufficient” or “not approved” in some regions. A full-scale reform of the Russian water industry and water security system is urgently needed.
